# Laboratory Response to Pandemic Threats: Challenges, Needs, and Solutions

**DOI:** 10.1089/hs.2022.0123

**Published:** 2023-09-27

**Authors:** Tyler Wolford, Jill Sutton, Chris N. Mangal

**Affiliations:** Tyler Wolford, MS, is Manager, Emergency Preparedness and Response, Association of Public Health Laboratories, Silver Spring, MD.; Jill Sutton is a Specialist, Emergency Preparedness and Response, Association of Public Health Laboratories, Silver Spring, MD.; Chris N. Mangal, MPH, is Director, Public Health Preparedness and Response, Association of Public Health Laboratories, Silver Spring, MD.

**Keywords:** COVID-19, Surveillance, Public health preparedness/response, Public health laboratory, Infectious diseases

## Introduction

The public health and healthcare sectors take different yet equally important approaches to serving and protecting the nation's health. At the foundation of both approaches is laboratory testing, which is necessary for health departments to monitor disease in the population and identify novel threats and for healthcare providers to make decisions to treat patients. The interdependency of public and private sector testing has never been more important, as seen with the response to SARS-CoV-2, the virus that caused the COVID-19 pandemic. Recent novel threats, such as COVID-19, compounded with reemerging diseases, such as mpox and Ebola, and environmental disasters, have strained laboratory systems across the globe.

In the United States, public health laboratories are governmental laboratories that perform 11 core functions (see Box). These laboratories collaborate with federal organizations, such as the US Centers for Disease Control and Prevention (CDC), US Environmental Protection Agency, US Food and Drug Administration, Federal Bureau of Investigation, Department of Homeland Security, and Department of Defense to prepare for and respond to public health events.^2^ In addition to federal partners, public health laboratories partner with private, hospital, and university laboratories, as well as first responders such as civil support teams and local law enforcement, to accomplish their mission of protecting the public's health.

In 2020, public health laboratories and their partners were called to action to provide testing services for COVID-19. This response stretched the capacity of the public health laboratories, especially given the initial challenges with developing and deploying tests. These delays in test development severely hindered the country's ability to detect and characterize transmission. Although the pandemic continues today, public health laboratories are using lessons learned to improve processes and plan for the future. Within each jurisdiction, after-action reviews are being conducted to identify challenges and needs, as well as to determine solutions to those issues.

The 2022 Preparedness Summit provided a unique opportunity for public health partners to come together and share their stories from the COVID-19 pandemic. At the Summit, the Association of Public Health Laboratories (APHL) cofacilitated a COVID-19 listening session with the Council of State and Territorial Epidemiologists. This listening session, titled Assessment and Surveillance, was dedicated to assessing current and next steps related to the assessment and surveillance of foundational public health capabilities.^3^ APHL was particularly concerned with identifying challenges public health laboratories faced during the COVID-19 response. Attendees highlighted the following laboratory-related challenges during the listening session:

- Need for expanded testing capability and capacity in laboratory response networks.
- Inability to sustain a skilled laboratory workforce, and gaps in developing crisis leaders.
- Need to strengthen partnerships and build new relationships with nontraditional partners.
- Lack of a dedicated supply chain for laboratories that is capable of meeting surge needs.

These issues are not new to public health laboratories and have been identified through various APHL-facilitated member forums and after-action reviews from previous responses (eg, 2009 H1N1 and the 2013 Ebola outbreaks). However, the sheer scale of the COVID-19 response, never before seen, further exposed these issues and reinforced the need to address these challenges to ensure a better-prepared public health laboratory system for the future.

Box 1. **Eleven Core Functions of Public Health Laboratories** ^1^

- Disease prevention, control, and surveillance
- Integrated data management
- Reference and specialized testing
- Environmental health and protection
- Food Safety
- Laboratory improvement and regulation
- Policy development
- Public health preparedness and response
- Public health-related research
- Training and education
- Partnerships and communication

## Challenges Faced by Public Health Laboratories

### Laboratory Response Networks

National laboratory networks are key to monitoring and rapidly detecting public health threats. In the United States, there are several networks that fill surveillance, detection, and response functions. Many public health laboratories are members of these various networks, including the Laboratory Response Network (LRN), Food Emergency Response Network, PulseNet, Environmental Response Laboratory Network, and the Global Influenza Surveillance Network, to name a few.

In previous responses to outbreaks, such as anthrax, Ebola, and recently mpox, CDC leveraged the LRN, which has 2 components—Biological Threats Preparedness (LRN-B) and Chemical Threats Preparedness (LRN-C)—with a third in development, Radiological Threats Preparedness (LRN-R). The biological threats preparedness component successfully responded to multiple threats since its creation in 1999 by APHL, CDC, and the Federal Bureau of Investigation.^4^ The LRN provides a link between state and local public health, federal, sentinel clinical, food, veterinary, environmental, agricultural, military, and international laboratories.^5^ The Department of Defense is an important LRN stakeholder, joining in strategic planning and assay development and supporting LRN-B response activities.^6^ While the LRN was initially designed to respond to bioterrorism, specifically anthrax, it has since expanded to include chemical threats and emerging infectious diseases. Given this expansion, it is unclear why CDC did not use the network to coordinate its response to COVID-19. It is important to note that public health laboratories at the local, state, and territorial levels used LRN assets, such as personnel and equipment, to respond to COVID-19, but national coordination, expected from the LRN, was not leveraged in this response.

A challenge with the substantial number of laboratory networks is the lack of coordination at the national level and a lack of awareness by multiple governmental agencies that they are essentially relying on the same laboratories for surge capacity. As CDC implements its Moving Forward strategy,^7^ it will be important to continue to elevate laboratory sciences within the agency and address the urgent need for a strategy for national laboratory coordination or, rather, a national laboratory system. The LRN should be seen as a vital asset to any national laboratory coordination plan. At the operational level, it will be imperative to maintain quality in surveillance and diagnostic assays while having a wide array of technologies positioned for deployment in a more integrated public–private laboratory system. Further, capabilities should continue to be modernized with automating extraction methods, optimizing methods for high-throughput testing on multiple platforms, and multiplexing assays. If not, the public health laboratory system will once again be strained in a large-scale response, evident after COVID-19 during the mpox response. In addition, standardized data exchange systems, such as Electronic Laboratory Reporting, should be used across public health programs to streamline data sharing and implementation of data reporting for emerging threats.

### Public Health Laboratory Workforce

As demonstrated by the testing needs of the COVID-19 pandemic, laboratories play a lead role in detection and response to infectious diseases, and that role cannot be performed without a highly trained and resilient workforce. During public health emergencies, laboratories must make staffing adjustments to ensure continuity of operations and meet testing demands. Further, public health laboratories play a pivotal role in training and outreach. For instance, during responses to novel threats, they are often consulted for guidance on biosafety and testing. Over the past 3 years, the COVID-19 pandemic continued to disrupt laboratory operations, impacting laboratory training, education, and outreach. Prolonged high testing volumes forced laboratories to divert staff for surge testing and required staff to work additional hours, which made it difficult to perform routine functions. As such, public health laboratories faced significant workforce challenges, including the inability to retain skilled personnel, staff burnout (both mental and physical), bureaucratic systems for recruitment, fear of political actions in jurisdictions where science was not valued, and a lack of a qualified pool of applicants. Another workforce challenge is the high turnover in leadership and a lack of skilled crisis response leaders.

Through a partnership between APHL and CDC, substantial funding has been provided to develop and execute the APHL Fellowship Program for public health laboratories.^8^ The fellowship program provides firsthand experience and training to various degree-level candidates in preparation for careers in public health laboratory science. The fellowships offered include disciplines such as bioinformatics, biorisk management, environmental health, food safety, infectious diseases, quality management, and newborn screening. APHL is currently developing a new fellowship for emergency preparedness and response focused on developing the crisis leadership skills and direct experience needed to effectively and efficiently lead through a public health response. Additionally, APHL provides leadership development opportunities via the APHL Emerging Leader Program, a 12-month program for laboratory professionals.^9^ The Emerging Leader Program provides the skills and experience to prepare graduates to transition into senior positions within their organizations. Along with promoting public health laboratory careers to the high school students, undergraduates, and the general public, fellowship programs and the Emerging Leader Program provide a strong platform to expand the skilled public health laboratory workforce.

While the 1-time funding to APHL will bolster the public health laboratory workforce, a sustained funding approach is needed to (1) introduce middle school and high students to public health laboratory science, (2) strengthen academic partnerships with universities and colleges to develop a pool of qualified personnel, (3) elevate the role of laboratory professionals and ultimately fortify recruitment, and (4) support professional development and other initiatives to retain highly skilled personnel.

### Partnerships

As is the case with emerging threats, effective and efficient laboratory testing is critical as it shapes treatment options and epidemiological actions, such as contact tracing, and influences larger public health decisions. Public health laboratories work closely with the private laboratory sector to prepare for and respond to existing and emerging public health threats. Supported primarily through funding from the federal government, namely CDC, public health laboratories lead efforts to develop and maintain partnerships with a diverse group of private and other governmental laboratories within their jurisdiction. In addition, commercial laboratories have an increasing role in surge support during a response. This was particularly important during the COVID-19 and mpox responses.

Recent responses highlighted the importance of expanding partnerships to nontraditional partners, such as nursing homes, schools, correctional facilities, and pharmacies. In many of these new partnerships, public health laboratories provided consultations, training on biosafety and performing risk assessments, and addressed sharing of test results. Public health laboratories were responsive to the needs of traditional and nontraditional partners during the COVID-19 pandemic, but the response highlighted the importance of formalizing relationships—that is, institution to institution—and having agreements in place that will outlive personnel transitions. An example of codifying partnerships is seen in the 2022 CDC Division of Laboratory Systems facilitation of the revised memorandum of understanding^10^ to address cooperation among several partners to enhance laboratory testing surge capacity outside of CDC and public health laboratories before and during a public health emergency. Partners from the Advanced Medical Technology Association, American Clinical Laboratory Association, Association for Molecular Pathology, APHL, College of American Pathologists, Council of State and Territorial Epidemiologists, US Food and Drug Administration, National Independent Laboratory Association, and CDC signed the memorandum of understanding.

### Dedicated Supply Chain for Laboratories

During an emergency response, laboratories increase testing capacity exponentially to support a surge in demand. This increase in testing and testers creates supply issues, especially when testing relies on 1 manufacturer to produce most consumables required for testing. During the COVID-19 pandemic, pipette tips were an example of a consumable that was difficult for public health laboratories to procure. Although pipette tips are manufactured by multiple vendors, the amount of testing being done completely exhausted the US supply. Public health laboratories were forced to determine alternate solutions at the risk of decreasing quality of testing. At the height of the COVID-19 response, public health laboratories voiced concerns about the lack of action by the federal government to solve supply chain issues. These laboratories noted that before and during future emergencies, it will be essential for the federal government to provide timely and consistent guidance, streamline the process for diagnostic development, and better restrict authorization of low-quality diagnostics, as this significantly impacts the supply chain. During the mpox response, public health laboratories in the LRN required specific manual nucleic acid extraction kits from 1 manufacturer to test using a US Food and Drug Administration 510(k) cleared assay. A limited supply of this extraction kit negatively impacted testing capacity, as this extraction kit was also being used by laboratories to support COVID-19 testing. Supply chain issues that arise during a response should be proactively addressed with strategic stockpile options or the ability of manufacturers to rapidly increase production to meet testing demand.

The Strategic National Stockpile is a federal program that provides medical countermeasures, such as supplies, medicines, and devices for state, local, tribal, and territorial jurisdictions in a public health emergency.^11^ Public health laboratories and partners would benefit from a similar program for nonperishable laboratory consumables during a public health emergency. A laboratory supply stockpile would require coordination with federal partners, vendors, and jurisdictions to fund, monitor, and ensure sufficient supply when needed. Partnerships with laboratory supply vendors are also needed to ensure perishable supplies, such as reagents, are made available to public health laboratories during a response.

## Conclusion

Public health laboratories are at the forefront of surveillance for threats, both novel and reemerging. These same laboratories also play a pivotal role in detecting all-hazard threats, performing outreach to traditional and nontraditional partners, providing guidance as appropriate, and evaluating new technologies. Over the years, technology to detect biological threat agents has evolved to include real-time polymerase chain reaction, matrix-assisted laser desorption/ionization time of flight mass spectrometry, and now, next-generation sequencing. Using next-generation sequencing technology, laboratorians collaborate closely with epidemiologists, bioinformaticians, and others to understand the transmission and evolution of pathogen outbreaks such as SARS-CoV-2. As we prepare for the next pandemic, these new technologies will be critical for accurate and timely detection of the threat.

As we look ahead, there is an urgent need for a national laboratory coordination strategy that clearly outlines a public–private laboratory system or network poised to detect the next threat. Such a strategy should also articulate the regulatory approach to ensuring quality diagnostics and a robust supply chain. Further, we cannot wait until the next pandemic to address the significant gaps facing the public health laboratory workforce and, more broadly, the laboratory workforce. A sustained funding model is needed to ensure a highly qualified cadre of laboratory personnel. At the local, state, and territorial levels, public health laboratory leaders must advocate for changes in their salary ranges and prioritize educating governmental officials on the educational and/or licensure requirements and duties of laboratory personnel.

A robust public health system is critical to respond to the plethora of threats—from environmental disasters to infectious diseases. With the reemergence of Ebola, Zika, mpox, and new threats such as SARS-CoV-2, timely and accurate laboratory testing is the linchpin to an effective response. The laboratory system, comprising private and public partners, must be in sync, working together to prioritize and respond to testing needs. In a postpandemic world, it is essential for the public health sector to rebuild partnerships and forge ahead with engaging new, nontraditional partners.
